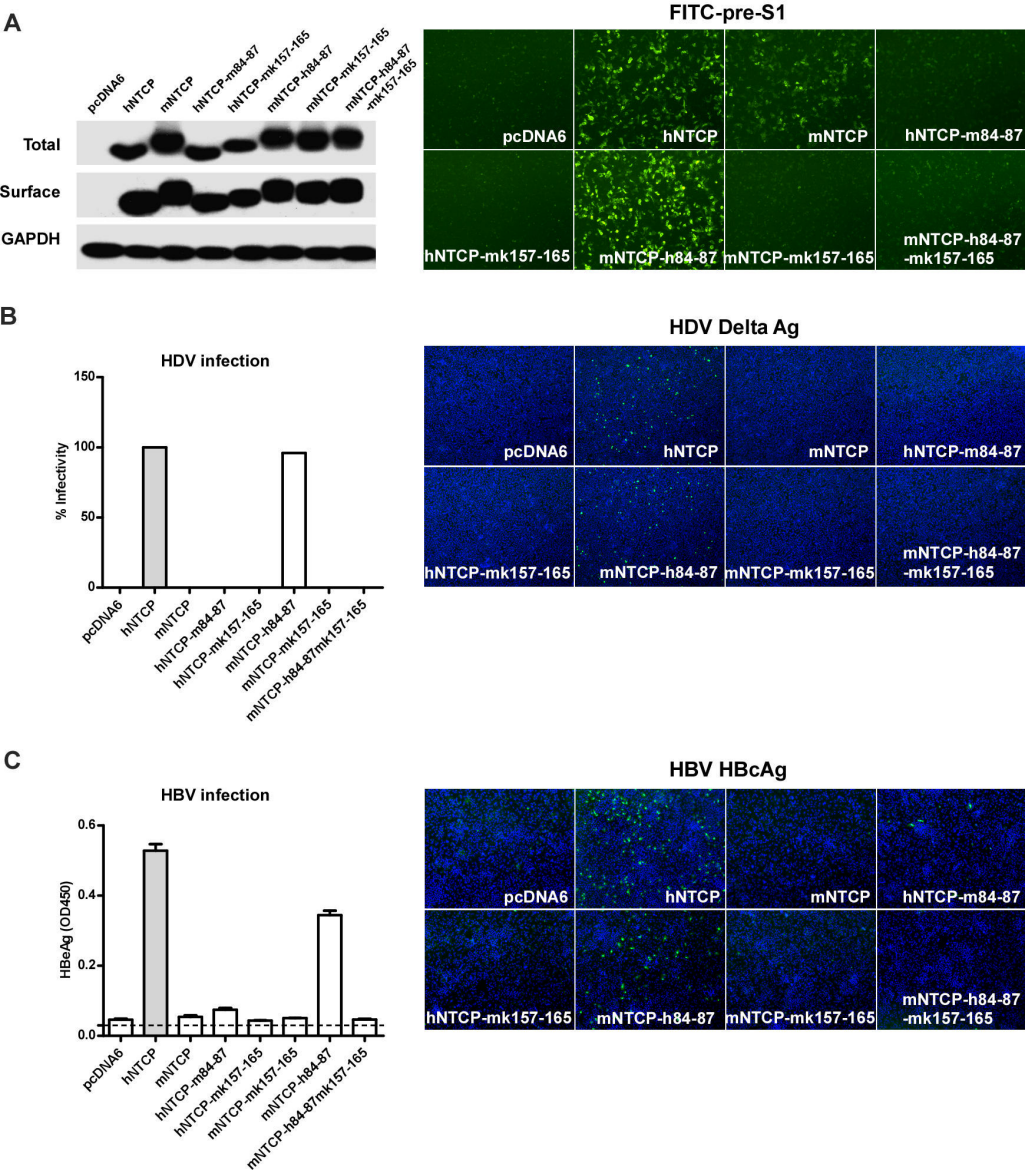# Correction for Yan et al., “Molecular Determinants of Hepatitis B and D Virus Entry Restriction in Mouse Sodium Taurocholate Cotransporting Polypeptide”

**DOI:** 10.1128/jvi.00445-25

**Published:** 2025-04-16

**Authors:** Huan Yan, Bo Peng, Wenhui He, Guocai Zhong, Yonghe Qi, Bijie Ren, Zhenchao Gao, Zhiyi Jing, Mei Song, Guangwei Xu, Jianhua Sui, Wenhui Li

## AUTHOR CORRECTION

Volume 87, no. 14, p. 7977–7991, 2013, https://doi.org/10.1128/jvi.03540-12. Page 7988: Figure 8A should appear as shown in this correction. In the original article, the two images on the far right (hNTCP-m84-87 and mNTCP-h84-87-mk157-165) were duplicates. This unfortunate duplication error probably occurred while preparing the two images for the figure and remained overlooked at later stages of the publication process. Both images show no binding of FITC-pre-S1 to the two mutated NTCP receptors.

Page 7988: Figure 8C should appear as shown in this correction. The label at the top of the left panel has been updated.

We sincerely apologize for the oversight. These errors have no impact on the conclusions.

**Fig 8:**